# Evidence for contrasting roles of dimethylsulfoniopropionate production in *Emiliania huxleyi* and *Thalassiosira oceanica*

**DOI:** 10.1111/nph.16374

**Published:** 2020-01-27

**Authors:** Erin L. McParland, Anna Wright, Kristin Art, Meagan He, Naomi M. Levine

**Affiliations:** ^1^ Department of Marine Chemistry and Geochemistry Woods Hole Oceanographic Institution Woods Hole MA 02543 USA; ^2^ Department of Marine and Environmental Biology University of Southern California Los Angeles CA 90089 USA

**Keywords:** dimethylsulfoniopropionate, *Emiliania huxleyi*, *F*_v_/*F*_m_, nitrate limitation, salinity stress, *Thalassiosira oceanica*, thermal curve

## Abstract

- Dimethylsulfoniopropionate (DMSP) is a globally abundant marine metabolite and a significant source of organic carbon and sulfur for marine microbial ecosystems with the potential to influence climate regulation. However, the physiological function of DMSP has remained enigmatic for >30 yr. Recent insight suggests that there are different physiological roles for DMSP based on the cellular DMSP concentrations in producers.
- Differential production of DMSP was tested with multiple physiological experiments that altered nitrate availability, salinity and temperature to create stressed growth and target different metabolic conditions in *Emiliania huxleyi*, a high DMSP producer and *Thalassiosira oceanica*, a low DMSP producer.
- *Emiliania huxleyi* intracellular DMSP did not respond to metabolically imbalanced conditions, while *Thalassiosira oceanica* intracellular DMSP was significantly correlated to stressed growth rate across all conditions tested and exhibited a plastic response on a timescale of hours in nonsteady‐state.
- The previous assumption that proposed DMSP mechanism(s) can be universally applied to all producers is shown to be unlikely. Rather, two distinct ecological roles for DMSP likely exist that differ by producer type, where: (1) the primary role of DMSP in high producers is a constitutive compatible solute; and (2) DMSP production in low producers is a finely tuned stress response.

## Introduction

Dimethylsulfoniopropionate (DMSP) is a globally abundant organic sulfur and carbon metabolite produced by a diverse array of organisms, from single‐celled marine prokaryotes and eukaryotes to macroalgae and brackish plants (van Diggelen *et al.*, 1986; Keller *et al.*, 1989; Van Alstyne & Puglisi, 2007; McParland & Levine, 2019). In particular, DMSP production in marine microbial eukaryotes has been confirmed in almost all major eukaryotic supergroups (McParland & Levine, 2019). DMSP can comprise up to 11% of cellular carbon in marine phytoplankton, and DMSP production accounts for as much as 5% of total primary production in both coastal and open ocean regimes (Stefels *et al.*, 2007; Galí *et al.*, 2013; Levine *et al.*, 2015). The pool of dissolved DMSP can be turned over multiple times per day, supplying up to 13% of the bacterial carbon demand and 100% of the bacterial sulfur demand, which suggests that this metabolite is a critical substrate for heterotrophic growth (Kiene *et al.,* 2000; Tripp *et al.,* 2008; Levine *et al.,* 2015). Indeed, some of the most abundant clades of marine heterotrophs, SAR11 and SAR86, cannot reduce sulfate and require organic sulfur compounds such as DMSP (Tripp *et al.*, 2008; Dupont *et al.*, 2012). Additionally, a DMSP degradation product, dimethylsulfide (DMS), is considered the most significant natural source of sulfur to the atmosphere and plays an important role in climate regulation as a source of cloud condensation nuclei (Charlson *et al.*, 1987; Lana *et al.*, 2012). Understanding the physiological function of DMSP in producers is critical for quantifying the significant role of DMSP in marine microbial ecosystem dynamics, global carbon cycling and climate.

**Figure 1:**
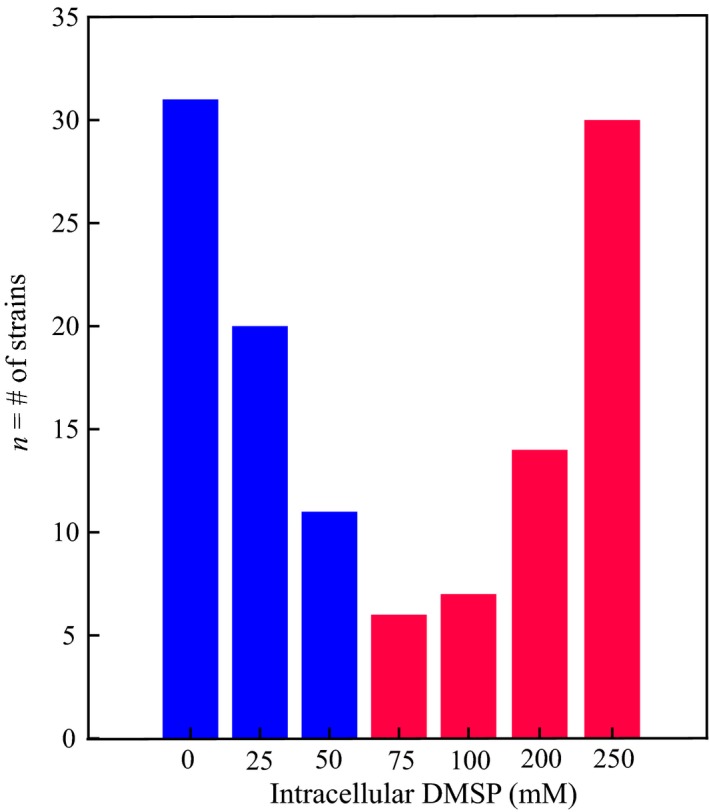
Histogram of intracellular dimethylsulfoniopropionate (DMSP) concentrations of previously measured strains grown in replete conditions. Colours represent the DMSP producer type (blue bars, low DMSP producers; red bars, high DMSP producers). Adapted from Supporting Information Table S1 in McParland & Levine (2019).

Intracellular DMSP concentrations span a wide range in eukaryotic producers from 0.01 to > 1000 mM (Keller *et al.*, 1989; Caruana & Malin, 2014; McParland & Levine, 2019). This distribution appears to be bi‐modal with two types of producers: high producers (HiDPs) with intracellular DMSP concentrations > 50 mM and low producers (LoDPs) with intracellular DMSP < 50 mM (Fig. 1). In general, prymnesiophytes and dinoflagellates are classified as HiDPs, and other important primary producers, such as diatoms and cyanobacteria, are typically LoDPs (Keller, 1989; Keller *et al.*, 1989; McParland & Levine, 2019). Current hypotheses for the cellular mechanism of DMSP production include its use as a compatible solute, a cryoprotectant, a ballasting mechanism, a signalling molecule, an overflow mechanism and an antioxidant (Karsten *et al.*, 1996; Stefels & Leeuwe, 1998; Stefels, 2000; Sunda *et al.*, 2002; Seymour *et al.*, 2010; Lavoie *et al.*, 2015; Johnson *et al.*, 2016). Most likely, DMSP plays multiple roles in the cell and/or different roles for different phytoplankton groups (Archer *et al.,* 2010; Bucciarelli *et al.,* 2013). However, despite over 30 yr of research, we currently lack an understanding of what these roles are and how they vary across different DMSP producers.

Experimental results from studies investigating the cellular mechanism of DMSP can be conflicting (van Rijssel & Buma, 2002; Archer *et al.,* 2010) and results that support a particular mechanism in a single strain are typically extrapolated to all DMSP producers. Furthermore, comparing across studies is complicated as measurements of changes in cellular concentration, rather than synthesis rates, are highly dependent on normalisation factors (e.g. cell number, cell carbon, or cell volume) (Keller, 1989; Stefels *et al.*, 2009). Previous studies have also compounded decreased and imbalanced growth, and steady‐state and nonsteady‐state conditions, making it difficult to distinguish potential mechanisms (McParland & Levine, 2019). A mechanistic understanding of the primary drivers behind DMSP production is essential for accurate predictions of *in situ* DMSP cycling, the sensitivity of the DMSP cycle to environmental changes, and the implications of these changes for marine ecosystems and the global climate.

Recently, a meta‐analysis of all previous monoculture studies of DMSP production proposed that HiDPs and LoDPs differentially regulate DMSP. Specifically, intracellular DMSP concentrations in HiDPs did not respond to nutrient limitation, suggesting that DMSP may be regulated constitutively in these producers. Intracellular DMSP in LoDPs significantly increased as a predictable function of nutrient limitation, suggesting that DMSP may be regulated as a stressed growth response in these producers (McParland & Levine, 2019). The two types of regulation were distinguished across a diverse range of DMSP producers (*n* = 20 strains) by classifying based on intracellular DMSP concentrations, not phylogeny. This contrasting behaviour of HiDPs and LoDPs was originally hypothesised by Stefels *et al.* (2007), but has never been tested by directly comparing HiDP and LoDP DMSP production under multiple conditions of stressed growth.

Here, we methodically compared changes in intracellular DMSP concentrations of a HiDP coccolithophore, *Emiliania huxleyi* and a LoDP diatom, *Thalassiosira oceanica*, in response to four different environmental conditions that uniquely altered cellular metabolism and growth rate: salinity stress, temperature stress, and nitrate limitation under steady‐state conditions, and nonsteady‐state nitrate limitation. The four conditions isolate the responses of DMSP production by the HiDP and LoDP to different low growth conditions, either metabolically balanced or imbalanced growth, with or without nutrient limitation. We also tested the plasticity of the DMSP mechanism with fine‐scale temporal measurements in nonsteady‐state. We confirmed the hypothesis of differential regulation under nutrient limitation proposed by McParland & Levine (2019) with the first direct comparison of a HiDP and LoDP and build on this hypothesis with observations of differential DMSP production across multiple metabolic conditions in steady‐ or nonsteady‐state. The results demonstrated that the two distinct groups of DMSP producers regulate DMSP differently, one as a constitutive function of cellular growth and the other as a function of stressed growth. Moreover, the different regulatory responses suggested that there are likely to be two very different physiological roles of DMSP. Critically, we propose that the current paradigm of viewing DMSP cycling through a single lens is incorrect, but rather DMSP is likely to function in two independent ecological cycles for which it serves different cellular mechanisms and responds to different ecological cues.

## Materials and Methods

### Culturing

Axenic cultures of *Thalassiosira oceanica* CCMP 1005 (*T. oceanica*) and *Emiliania huxleyi* CCMP 373 (*E. huxleyi*) were obtained from the National Center for Marine Algae and Microbiota. Cultures were grown in f/25 medium made with natural seawater (collected at the San Pedro Ocean Time‐series station, 33°33′N, 118°24′W). 0.2 µm filtrate of natural seawater was incubated in the dark for > 2 wk, microwave sterilised, and then nutrients were added ([NO_3_^−^] = 100 µM, [PO_4_^−^] = 6.25 µM, all other nutrients at f/2 concentrations) (Iglesias‐Rodriguez *et al.,* 2008). Cultures were maintained semi‐continuously in 1 l polycarbonate bottles at 23°C under fluorescent light (100 µmol photons m^−2^ s^−1^, 14 h : 10 h, light : dark cycle). Culture flasks were gently shaken by hand at least once per day to avoid CO_2_ limitation (Bochenek *et al.*, 2013).

For all steady‐state experiments, *in vivo* fluorescence was used to semi‐continuously acclimate each species to the experimental condition in triplicate until growth rates remained constant across three transfers (Wood *et al.*, 2005). After the third transfer, the bottles were sampled for cell counts, cellular biovolume, Chlorophyll *a* (Chl*a*), *F*_v_/*F*_m_, and total DMSP in mid‐exponential phase and again 24 h later. Maintenance culturing conditions were altered as follows to create different types of steady‐state stressed growth. Experiment 1: Each species was grown semi‐continuously under maintenance conditions in water baths set to 14°C, 16°C, 20°C, 23°C, 26°C or 28°C. Experiment 2: Each species was grown semi‐continuously under maintenance conditions in low nitrate f/25 medium ([NO_3_^−^] = 8 µM). Two low nitrate conditions were assessed: one in which each species was semi‐continuously acclimated to low nitrate in mid‐exponential phase and one in which each species was semi‐continuously acclimated to low nitrate in late exponential phase. Experiment 3: Media of different salinities were made by mixing a hypersaline solution (65‰) of ESAW artificial salts (Harrison & Berges, 2005) with either 0.2 µm filter sterile seawater or MilliQ water. Media with final salinities of 25‰, 35‰, 40‰ and 50‰ for *T. oceanica* and 25‰, 30‰, 35‰, 40‰ and 45‰ for *E. huxleyi* were confirmed with a refractometer after microwave sterilisation. Nutrients were added at maintenance concentrations (replete). By diluting all salts, these experiments altered not only salinity, but also the availability of sulfate, which has been shown previously to alter DMSP concentrations in *E. huxleyi*, but not in *Thalassiosira pseudonana* (Bochenek *et al.*, 2013; Kettles *et al.*, 2014).

Steady‐state low nitrate acclimated cultures of both species were used to quantify the nonsteady‐state response to the alleviation of nitrate limitation. Experiment 4: The experiment was started in mid‐exponential phase by adding back nitrate to replete concentrations ([NO_3_^−^] = 100 µM). Control cultures remained in low nitrate conditions ([NO_3_^−^] = 8 µM). Both conditions were conducted in triplicate. Samples for cell counts, cellular biovolume, chlorophyll *a*, *F*_v_/*F*_m_, and total DMSP were collected at 0, 1, 3, 6, 9, 12 and 24 h after nitrate add‐back.

### DMSP

Culture bottles were gently inverted three times before sampling for total DMSP (DMSPt). Unfiltered culture (10 ml) was collected in Falcon tubes and immediately acidified to pH 2 with 50% H_2_SO_4_ (3.3 µl per 1 ml of sample) (Kiene & Slezak, 2006). DMSPt samples were stored for a minimum of 24 h to allow for the complete oxidation of DMS (del Valle *et al.*, 2011). An aliquot of sample (1 ml for *E. huxleyi*, 3 ml for *T. oceanica*) was dispensed into an acid‐washed, combusted 14 ml serum vial with 1 ml of 5 N NaOH and crimped closed with a gas‐tight Teflon lid. Samples were vortexed and incubated for 10 min to cleave DMSP to DMS via alkaline hydrolysis (Kiene & Service, 1991). DMSP (derivatised to DMS) was quantified via headspace analysis using a gas‐tight Hamilton syringe with a custom Shimadzu 2016 gas chromatograph with a flame photometric detector and a Chromosil 330 packed column (Supelco, Bellefonte, PA, USA). Column temperature was 70°C and retention time was 0.92 min. DMSP concentrations were calculated with a four‐point DMS standard curve (*R* ^2^ > 0.95). Experimental triplicates and technical duplicates (*n* = 6 per treatment) were quantified. DMSPt concentrations were calculated as nmoles DMSPt per l culture (nM). Intracellular DMSP concentrations were calculated as mmoles DMSPt per l of cell volume^−1^ (mM).

### *F*_v_/*F*_m_

*F*_v_/*F*_m_ is the maximum quantum yield of photosystem II (PSII) (Butler, 1978) and was measured with a WALZ Phyto‐PAM. Unfiltered culture (3 ml) was collected in duplicate and dark adapted for 20 min (Kolber *et al.*, 1988, 1990). *F*_o_ was measured under low frequency (25 Hz) pulses and *F*_m_ was measured with a saturating pulse of 2600 µmol m^−2^ s^−1^ for 200 ms. *F*_v_/*F*_m_ was calculated as:

$$F_{v}/F_{m}=\frac{F_{m}-F_{o}}{F_{m}}.$$

### Ancillary measurements

Chlorophyll *a* (Chl*a*) was measured by gently vacuum filtering 5–10 ml of culture onto a 25 mm Whatman GF/F filter. Filters were stored at −20°C until extraction in 2.5 ml of 90% HPLC‐grade acetone within 36 h of collection. Samples were analysed using a Turner Trilogy fluorometer (Welschemeyer, 1994). Cell counts were quantified using 1.5 ml of culture preserved with 20 µl of 37% formaldehyde (final concentration = 0.5%). Cell counts were then enumerated with light microscopy (Zeiss) using a 0.1 mm Neubauer haemacytometer (Hausser Scientific, Horsham, PA, USA). Cell images were taken using a Zeiss AxioCam MRc 5 at ×400 magnification and dimensions were measured using Matlab software (diameter for *E. huxleyi* and diameter and height for *T. oceanica*). Numbers of pixels along a straight line were converted to µm using an image of a stage micrometer for calibration. Cell biovolumes were calculated using the geometric volume formula of a sphere for *E. huxleyi* and a cylinder for *T. oceanica*. Cellular biovolumes for each species were used to calculate intracellular DMSP (Supporting Information Table S1). Finally, growth rates were calculated as:

$$\mu =\frac{\log_{e}\left(X_{f}\right)-\log_{e}\left(X_{i}\right)}{t},$$

where *X*_f_ is cell concentration at the final time point, *X*_i_ is cell concentration at the initial time point, and *t* is the interval between the measurements in days.

### Cellular osmolarity calculations

Medium osmolarity was calculated as described in Boyd & Gradmann (2002). Total cellular osmolarity was assumed to be maintained 20% higher than the medium osmolarity and equal to the sum of ionic osmolarity and organic osmolarity (Sikes & Wilbur, 1982; Lavoie *et al.*, 2015). Organic osmolarity was calculated as the difference of total cellular osmolarity minus total ionic osmolarity. Ionic osmolarity was assumed based on a previous study (Lavoie *et al.*, 2015).

Previously measured ionic and organic osmolarity varies widely across species (Keller *et al.*, 1999a; Boyd & Gradmann, 2002; Gebser & Pohnert, 2013). Therefore, we used the species‐specific ionic osmolarities previously estimated for *E. huxleyi* and *Thalassiosira pseudonana* (Lavoie *et al.,* 2015). The ionic osmolarity was estimated by Lavoie *et al.* (2015) for each species based on previously published literature values of intracellular concentrations of the major inorganic ions and known ratios of intracellular inorganic ions and/or total cellular osmolarity. We also assumed that cellular concentrations of major ions linearly scaled with changes in external osmolarity (Dickson & Kirst, 1987; Table S2). The DMSP contribution is presented as a percent of the calculated total organic osmolarity.

### Statistical tests

We report all errors as the error propagation of the standard deviation of biological triplicates within technical replicates. For *E. huxleyi* grown under steady‐state hyposaline conditions (25‰) and low nitrate in late exponential phase, the reported error reflected biological duplicates as the third replicate bottles in these experiments crashed.

For all steady‐state experiments, significance between treatments was tested using the two‐tailed Student's *t*‐test. Correlations between measured variables were tested using a linear regression. In Experiment 4, a two‐way repeated measures analysis of variance (ANOVA) with the factors time and treatment (with or without NO_3_^−^ add‐back) was performed to account for dependent sampling. All statistical tests were performed using matlab and a *P*‐value ≤ 0.05 was considered significant. Results are reported as a single *P*‐value, *R* ^2^ value and *P*‐value or *F*‐value and *P*‐value when a *t*‐test, linear regression or repeated measures ANOVA was performed, respectively.

## Results

We quantified DMSP production by a HiDP (*E. huxleyi*) and a LoDP (*T. oceanica*) simultaneously with growth rate (µ), PSII efficiency (*F*_v_/*F*_m_), and cellular Chl*a* under four conditions of stressed growth (Table 1). We also estimated the potential osmotic contribution of DMSP as the per cent contribution of DMSP to cellular organic osmolarity. As the mass balance of cellular osmolarity is still not known (Raven & Doblin, 2014), we use species‐specific estimates of ionic osmolarity and assumed the remaining osmotic balance was met with DMSP and other organic osmolytes. Based on the previous observation of two consistently different responses of DMSP production to nutrient limitation in multiple strains of LoDPs and HiDPs (*n* = 11 and *n* = 9 strains, respectively) (McParland & Levine, 2019), we expected that the responses of the two model DMSP producers used here would reflect general differences between all LoDPs and HiDPs.

**Table 1:** Summary of experimental conditions, associated metabolic conditions and the intracellular dimethylsulfoniopropionate (DMSP) response by *Emiliania huxleyi* and *Thalassiosira oceanica*.

Treatment	Condition	Metabolic state	HiDP DMSP (mM)	LoDP DMSP (mM)
Temperature	≤ *T*_opt_	Balanced	↑	↑
	> *T*_opt_	Imbalanced	–	NA
Nitrate limited	Semi‐continuous	Imbalanced	–	↑
Salinity	Hyposaline (≤ opt salinity)	Unknown	–	NA
	Hypersaline (> opt salinity)	Unknown	↑	↑

↑, increased intracellular DMSP; −, no significant change in intracellular DMSP; HiDP, high producers; LoDP, low producers; NA, intracellular DMSP could not be measured.

The experiments were designed to isolate differential responses by *E. huxleyi* and *T. oceanica* under metabolically balanced and imbalanced growth (Table 1). Previous studies have demonstrated that growth at temperatures below the optimal growth temperature (*T*_opt_) is metabolically balanced, with similar decreases in photosynthesis and respiration (Thomas *et al.*, 2012; Baker *et al.*, 2016; Barton *et al.*, 2018). By contrast, growth at temperatures above *T*_opt_ and under nutrient limitation (steady‐ or nonsteady‐state) was linked to metabolic imbalances, in which photosynthesis and respiration are decoupled (Hockin *et al.,* 2012; Baker *et al.,* 2016; Wordenweber *et al.,* 2018). The metabolic conditions of marine phytoplankton resulting from steady‐state hyper‐ and hyposaline stresses are relatively unknown, as most previous studies have focused on euryhaline species or nonsteady‐state responses to osmotic shock (Qasim *et al.*, 1972; Macler, 1988; Jahnke & White, 2003; Bussard *et al.*, 2017). Extreme salinity stress is however well known to induce oxidative stress (Jahnke & White, 2003; Acosta‐Motos *et al.*, 2017).

### Temperature stress (balanced and imbalanced growth)

*E. huxleyi* and *T. oceanica* were grown in steady‐state under nutrient replete conditions across a thermal gradient. Both *E. huxleyi* and *T. oceanica* growth exhibited classic, skewed, thermal response curves with a *T*_opt_ of 23°C and 26°C, respectively (Fig. S1; Table 2). We compared responses to temperatures ≤*T*_opt_ (balanced growth) and > *T*_opt_ (imbalanced growth). For both species under balanced low growth (≤ *T*_opt_), *F*_v_/*F*_m_ was positively correlated with increasing temperature (*R* ^2^ > 0.7, *P* ≤ 0.05) (Fig. S2a,b) and cellular Chl*a* was significantly higher than at *T*_opt_ (*P* ≤ 0.05) (Table 2). For *E. huxleyi* at temperatures > *T*_opt_, *F*_v_/*F*_m_ continued to increase (Fig. S2a) and cellular Chl*a* was slightly higher at 28°C than at *T*_opt_ (*P* = 0.07) (Table 2). The thermal response curve for *T. oceanica* was more skewed than for *E. huxleyi*: µ at 28°C was not significantly lower than µ at *T*_opt_ (*P* = 0.7) and zero growth was observed at 30°C (Fig. S1b). Therefore, the response of *T. oceanica* grown at temperatures > *T*_opt_ could not be quantified.

**Table 2:** Mean ± error of µ, *F*_v_/*F*_m_, cellular Chl*a*, and intracellular dimethylsulfoniopropionate (DMSP) for *Emiliania huxleyi* and *Thalassiosira oceanica* in all steady‐state experiments.

	*E. huxleyi*								*T. oceanica*							
	μ (d^−1^)	±	*F*_v_/*F*_m_	±	Chl*a* per cell (μg cell^−1^)	±	Intracellular DMSP (mM)	±	μ (d^−1^)	±	*F*_v_/*F*_m_	±	Chl*a* per cell (μg cell^−1^)	±	Intracellular DMSP (mM)	±
Temperature stress																
< *T*_opt_ (14°C)	0.3	0.1*	0.59	0.005*	3.6 × 10^−7^	3.6 × 10^−8^ *	323	50*	0.3	0.1*	0.57	0.000*	5.0 × 10^−7^	8.4 × 10^−8^ ns	10	2*
< *T*_opt_ (16°C)	0.3	0.1*	0.59	0.005*	3.6 × 10^−7^	4.4 × 10^−8^ *	307	49*	0.4	0.0*	0.57	0.003*	5.0 × 10^−7^	6.9 × 10^−8^ *	9	1*
< *T*_opt_ (20°C)	0.8	0.1 ns	0.65	0.002*	2.9 × 10^−7^	2.1 × 10^−8^ ns	198	26*	0.5	0.1*	0.58	0.004*	5.5 × 10^−7^	8.8 × 10^−8^ *	8	1*
Control: *T*_opt_ (23°C)	0.9	0.1	0.63	0.000	2.6 × 10^−7^	1.9 × 10^−8^	145	19	0.6	0.0*	0.61	0.004*	5.8 × 10^−7^	8.8 × 10^−8^ *	7	1*
> *T*_opt_ (26°C)	0.5	0.2*	0.71	0.003*	1.9 × 10^−7^	1.6 × 10^−8^ *	67	9*	0.8	0.1	0.69	0.000	4.3 × 10^−7^	4.2 × 10^−8^	2	0.2
> *T*_opt_ (28°C)	0.3	0.3*	0.68	0.002*	3.6 × 10^−7^	3.6 × 10^−8^ ns	119	17 ns	0.8	0.1 ns	0.70	0.005 ns	3.7 × 10^−7^	2.9 × 10^−8^ ns	2	0.2*
NO_3_^−^ stress																
Control: N_ss_^+^	0.9	0.0	0.68	0.003	3.0 × 10^−7^	5.1 × 10^−8^	165	23	1.0	0.2	0.69	0.003	3.9 × 10^−7^	3.0 × 10^−8^	4	0.3
N_ss_^−^	0.6	0.1*	0.70	0.002*	3.1 × 10^−7^	4.0 × 10^−8^ ns	132	24*	0.7	0.0*	0.69	0.004 ns	2.8 × 10^−7^	6.3 × 10^−9^ *	8	0.3*
N_ss_^−−^	0.3	0.0*	0.44	0.000*	2.7 × 10^−7^	5.5 × 10^−8^ ns	146	36 ns	0.3	0.0*	0.64	0.003*	2.6 × 10^−7^	9.8 × 10^−9^ *	12	0.5*
Salinity stress																
< Opt salinity (25‰)	0.1	0.0*	0.64	0.000 ns	1.3 × 10^−7^	1.2 × 10^−8^ *	48	6 ns	0.0	–	–	–	–	–	–	–
< Opt salinity (30‰)	0.8	0.0 ns	0.64	0.000 ns	1.0 × 10^−7^	1.1 × 10^−8^ ns	50	8 ns	–	–	–	–	–	–	–	–
Control: opt salinity (35‰)	0.7	0.0	0.63	0.000	9.4 × 10^−8^	1.2 × 10^−8^	57	10	1.4	0.2	0.61	0.002	2.3 × 10^−7^	1.9 × 10^−8^	0.9	0.1
> Opt salinity (40‰)	0.5	0.1 ns	0.63	0.005 ns	1.0 × 10^−7^	1.2 × 10^−8^ ns	82	13*	1.3	0.2 ns	0.64	0.002*	2.9 × 10^−7^	2.9 × 10^−8^ *	4	0.4*
> Opt salinity (45‰ or 50%)	0.2	0.1*	0.58	0.002*	1.1 × 10^−7^	1.9 × 10^−8^ ns	133	28*	0.2	0.0*	0.42	0.002*	3.0 × 10^−7^	2.0 × 10^−8^ *	14	1*

ns, not significant.

* Treatment is significantly different than control (*P* ≤ 0.05).

Error bars represent ± SD.

**Figure 2:**
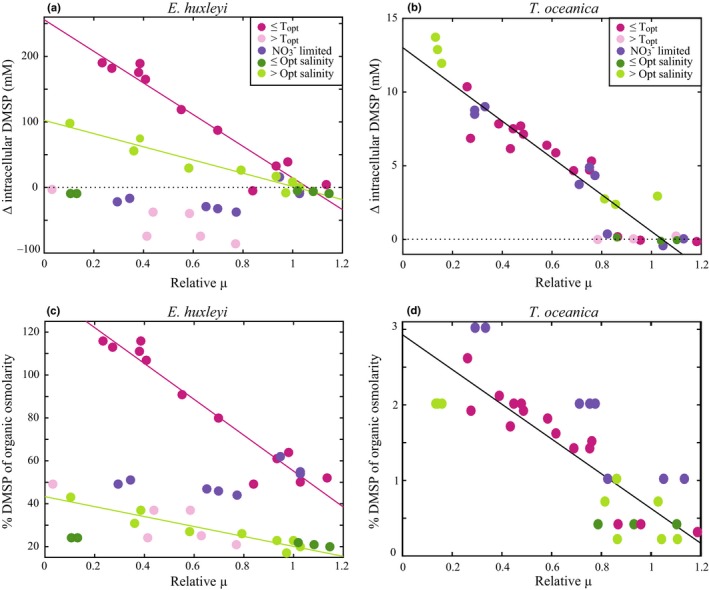
Change in intracellular dimethylsulfoniopropionate concentrations (∆ intracellular DMSP) and the predicted percent contribution of DMSP to organic osmolarity versus relative fold change in growth rate (relative µ) for *Emiliania huxleyi* (a, c) and *Thalassiosira oceanica* (b, d) in all steady‐state experiments. ∆ intracellular DMSP represents the difference between treatment and control. Relative µ represents fold change in treatment µ relative to control µ (0, no growth). Colours reflect the conditions of ≤ optimal temperature (*T*_opt_), > *T*_opt_, NO_3_^−^ limited, ≤ optimal salinity (hyposaline) and > optimal salinity (hypersaline). Black dotted line represents no change in intracellular DMSP (a, b). Solid lines are significant linear regressions for *E. huxleyi* ≤ *T*_opt_ and hypersaline experiments (a, c) and for *T. oceanica* across all steady‐state experiments (b, d).

Intracellular DMSP at *T*_opt_ was 145 ± 19 mM in *E. huxleyi* and 2 ± 0.2 mM in *T. oceanica* (Table 2), which is consistent with previous measurements of the same strains (Steinke *et al.*, 1998; Arnold *et al.*, 2013; Bucciarelli *et al.*, 2013). Under balanced low growth (≤ *T*_opt_), intracellular DMSP was significantly negatively correlated with µ in both *E. huxleyi* and *T. oceanica* (*R* ^2^ > 0.8, *P* ≤ 0.05) (Fig. 2a,b). Specifically, over a three‐fold decrease in µ (*c.* 0.9 to 0.3 d^−1^), intracellular DMSP increased two‐fold and six‐fold for *E. huxleyi* and *T. oceanica*, respectively (Fig. S3a,b; Table 2). Under imbalanced low growth (> *T*_opt_), *E. huxleyi* intracellular DMSP became decoupled from µ as intracellular DMSP was either lower than or unchanged from the concentration at *T*_opt_ (Figs 2a, S3a; Table 2).

### Nitrate limitation (imbalanced growth)

*E. huxleyi* and *T. oceanica* were grown in steady#x2010;state under three different nitrate (NO_3_^−^) conditions: NO_3_^−^ replete in exponential phase (N_ss_^+^), NO_3_^−^ limited in mid‐exponential phase (N_ss_^−^) and NO_3_^−^ limited in late exponential phase (N_ss_^−−^). Both conditions of NO_3_^−^ limitation resulted in imbalanced growth conditions. µ significantly decreased with increasing NO_3_^−^ limitation in both species (*P* ≤ 0.05) (Table 2). In *E. huxleyi*, *F*_v_/*F*_m_ significantly decreased in N_ss_^−−^ (Δ *F*_v_/*F*_m_ = 0.24) (*P* ≤ 0.05) (Fig. S2c), while cellular Chl*a* remained unchanged (*P* = 0.9) (Table 2). This suggests that PSII efficiency decreased under NO_3_^−^ limitation in *E. huxleyi*, even though cellular Chl*a* was maintained at optimal concentrations (unchanged relative to N_ss_^+^). In *T. oceanica*, both *F*_v_/*F*_m_ and cellular Chl*a* significantly decreased in N_ss_^−−^ (*P* ≤ 0.05) (Fig. S2d; Table 2). Although the observed *F*_v_/*F*_m_ changes were significant for both species, the magnitude of change was small in *T. oceanica* (Δ *F*_v_/*F*_m_ = 0.05). We confirmed the negative relationship between *F*_v_/*F*_m_ and NO_3_^−^ limitation in *T. oceanica* with additional measurements (Fig. S4), which further suggested that these small changes in *F*_v_/*F*_m_ reflected the cellular response to the imbalanced growth conditions of NO_3_^−^ limitation.

*E. huxleyi* intracellular DMSP remained high with no significant changes in N_ss_^−−^ (165 ± 23 mM in N_ss_^+^ vs 146 ± 36 mM in N_ss_^−−^) (*P* = 0.2) (Figs 2a, S3c). By contrast, *T. oceanica* intracellular DMSP linearly increased with increasing NO_3_^−^ limitation from 4 ± 0.3 mM in N_ss_^+^ to 12 ± 0.5 mM in N_ss_^−−^ (*R* ^2^ = 0.9, *P* ≤ 0.05) (Fig. 2b). Specifically, as µ decreased three‐fold in *T. oceanica* from N_ss_^+^ to N_ss_^−−^, intracellular DMSP increased three‐fold.

### Salinity stress

*E. huxleyi* and *T. oceanica* were grown in a steady‐state under nutrient replete conditions across a salinity gradient. While the metabolic conditions of these species under steady‐state hyper‐ and hyposaline stresses are unknown (Table 1), salinity changes significantly decreased µ in both species relative to µ at optimal salinity (35‰). Hypersaline conditions decreased µ four‐fold in *E. huxleyi* and seven‐fold in *T. oceanica*, and hyposaline conditions decreased µ seven‐fold in *E. huxleyi*. As no growth was observed for *T. oceanica* under hyposaline conditions (Table 2), the response could not be tested. Similar to the NO_3_^−^ limitation response, cellular Chl*a* in *E. huxleyi* did not change significantly under hypersaline conditions (*P* = 0.1) (Table 2), but *F*_v_/*F*_m_ exhibited a small, significant decrease relative to optimal salinity (Δ *F*_v_/*F*_m_ = 0.05) (*P* ≤ 0.05) (Fig. S2e). In hyposaline conditions, cellular Chl*a* in *E. huxleyi* significantly increased (*P* ≤ 0.05) but no significant change was observed in *F*_v_/*F*_m_ (*P* = 0.2). In *T. oceanica* under hypersaline conditions, cellular Chl*a* significantly increased, while *F*_v_/*F*_m_ significantly decreased (Δ*F*_v_/*F*_m_ = 0.2) (*P* ≤ 0.05) (Fig. S2f; Table 2).

For both species in hypersaline conditions, intracellular DMSP was significantly positively correlated with increasing salinity and negatively correlated with µ (*R* ^2^ > 0.9, *P* ≤ 0.05) (Figs 2a,b, S3e,f). The increase in *E. huxleyi* intracellular DMSP more than compensated for the increased osmotic demand, with the predicted contribution of DMSP to organic osmolarity increasing from 20% to 37% in hypersaline conditions (Fig. 2c). A small increase in the contribution of DMSP to organic osmolarity from 0.2% to 2% was also predicted for *T. oceanica* in hypersaline conditions (Fig. 2d). While assumptions of ionic osmolarity introduce error in the exact estimates of DMSP contribution to organic osmolarity, we expect the magnitudes and relative changes of DMSP contribution to osmolarity in response to the environmental stressors presented here to be unaffected by this uncertainty.

### All steady‐state

Depressed growth rates in *T. oceanica* resulted in increased intracellular DMSP across all steady‐state experiments. Critically, the same linear relationship between µ and intracellular DMSP was observed across all conditions (*R* ^2^ = 0.8, *P* ≤ 0.05) (Fig. 2b). The greatest fold decrease in µ relative to the control in hypersaline conditions (50‰) resulted in the greatest increase in intracellular DMSP and subsequently the greatest contribution to organic osmolarity (Fig. 2b,d). This constant relationship between DMSP and growth suggests that *T. oceanica* produced DMSP as a function of stressed growth, independent of the stressor type or different metabolic conditions associated with each (Fig. 2b). *E. huxleyi* exhibited a very different, and noisier, relationship between µ and intracellular DMSP (Fig. 2a). Changes in intracellular DMSP in *E. huxleyi* were significantly correlated to µ in hypersaline stress and temperatures ≤ *T*_opt_ (*R* ^2^ = 0.8, *P* ≤ 0.05), but showed no relationship to µ under the imbalanced metabolic conditions of NO_3_^−^ limitation and temperatures > *T*_opt_ (*R* ^2^ < 0.6, *P* > 0.05) (Fig. 2a). Under hypersaline stress, however, the increase in *E. huxleyi* intracellular DMSP could be attributed to the significant cellular osmotic adjustments in response to medium osmolarity changes (Fig. 2c). Therefore, the only significant change in *E. huxleyi* intracellular DMSP concentrations, not directly linked to osmolarity changes, occurred under temperatures ≤ *T*_opt_ (Fig. 2a).

### Nonsteady‐state nitrate add‐back (imbalanced growth)

**Figure 3:**
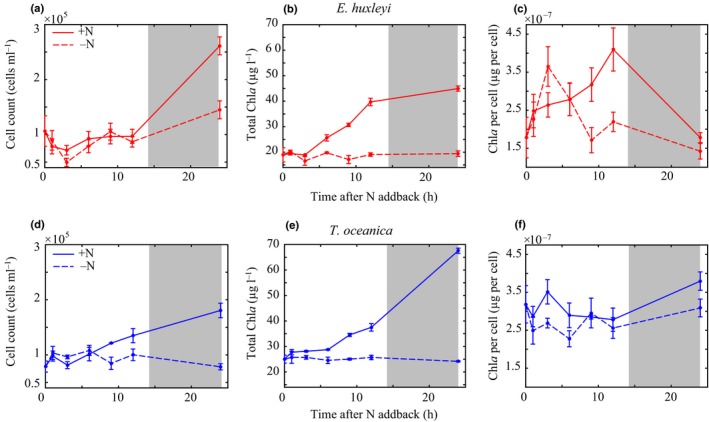
Cell counts, total Chl*a*, and Chl*a* per cell after NO_3_^−^ add‐back for *Emiliania huxleyi* (a–c) and *Thalassiosira oceanica* (d–f). Solid lines indicate the NO_3_^−^ add‐back treatment (+N). Dashed lines represent the control treatment (−N). Grey shading indicates the dark period (14 h : 10 h, light : dark cycle). Error bars represent ± SD.

The plastic response (nonsteady‐state) was quantified by tracking intracellular DMSP changes in *E. huxleyi* and *T. oceanica* for 24 h after alleviation of NO_3_^−^ limitation in N_ss_^−^ cultures (+N). Control cultures were maintained in N_ss_^−^ (−N). Both Chl*a* (*F*_1,24_ = 96 and *F*_1,24_ = 136, *P* ≤ 0.05) and cell concentrations (*F*_1,24_ = 47 and *F*_1,24_ = 9, *P* ≤ 0.05) significantly increased in +N *T. oceanica* and *E. huxleyi* after 24 h, confirming that NO_3_^−^ limited growth before the NO_3_^−^ addition (Fig. 3).

The time course of Chl*a* and cell concentrations in response to the NO_3_^−^ add‐back differed between species (Fig. 3). Chl*a* significantly increased in the first 12 h in +N *E. huxleyi* following the NO_3_^−^ add‐back (*F*_1,8_ = 152, *P* ≤ 0.05), while cell concentrations remained constant (Fig. 3c). Cell concentrations then significantly increased between 12 and 24 h in +N (*F*_1,8_ = 11, *P* ≤ 0.05), indicating that *E. huxleyi* divided during this time period (Fig. 3b). Other than the deviation before cell growth (time points 3–12 h), *E. huxleyi* cellular Chl*a* concentrations were not statistically different in +N and −N cultures (*F*_1,8_ = 0.8, *P* = 0.3) (Fig. 3c). By contrast, +N *T. oceanica* responded rapidly to the NO_3_^−^ add‐back by increasing both Chl*a* and cell concentrations continuously over the 24 h (*F*_1,28_ = 96 and *F*_1,28_ = 47, *P* ≤ 0.05), with cell concentrations significantly higher than –N beginning at 9 h (*F*_1,8_ = 72, *P* ≤ 0.05). After 24 h, cellular Chl*a* in *T. oceanica* was statistically higher in +N conditions than in −N (*F*_1,28_ = 10, *P* ≤ 0.05) and was similar to steady‐state NO_3_^−^ replete cellular Chl*a* (Fig. 3f; Table 2).

**Figure 4:**
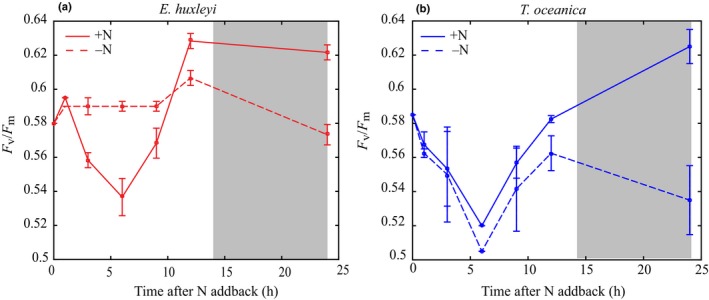
*F*_v_/*F*_m_ after NO_3_^−^ add‐back for *Emiliania huxleyi* (a) and *Thalassiosira oceanica* (b). Solid lines indicate the NO_3_^−^ add‐back treatment (+N). Dashed lines represent the control treatment (−N). Grey shading indicates the dark period (14 h : 10 h, light : dark cycle). Error bars represent ± SD.

Despite the differences in cellular Chl*a* and cell division, changes in *F*_v_/*F*_m_ over the experiment were consistent for *E. huxleyi* and *T. oceanica* (Fig. 4). In both species after 24 h, a small but significant increase in *F*_v_/*F*_m_ was observed in +N cultures (Δ*F*_v_/*F*_m_ = 0.03 for *E. huxleyi* and 0.04 for *T. oceanica*) relative to −N (*F*_1,8_ = 42 and *F*_1,8_ = 16, *P* ≤ 0.05). Each species exhibited a unique diel feature at the midpoint of the light cycle, but these features were determined not to have significant implications for DMSP production and are therefore only discussed in the Supporting Information (Notes S1; Fig. S5). *F*_v_/*F*_m_ in both species was significantly correlated with total Chl*a* concentrations (*R* ^2 ^> 0.5, *P* ≤ 0.05) (Figs 3, 4), consistent with the response of *F*_v_/*F*_m_ to NO_3_^−^ limitation observed in the steady‐state experiments.

**Figure 5:**
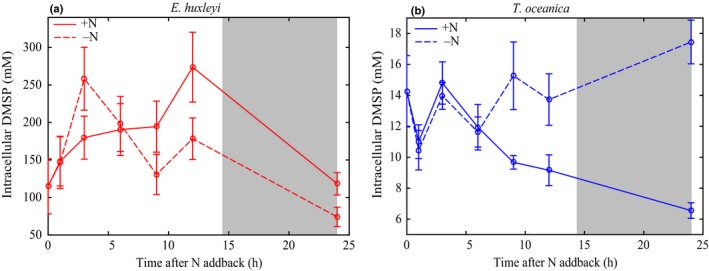
Intracellular dimethylsulfoniopropionate (DMSP) after NO_3_^−^ add‐back for *Emiliania huxleyi* (a) and *Thalassiosira oceanica* (b). Solid lines indicate the NO_3_^−^ add‐back treatment (+N). Dashed lines represent the control treatment (−N). Grey shading indicates the dark period (14 h : 10 h, light : dark cycle). Error bars represent ± SD.

While DMSPt significantly increased in the +N *E. huxleyi* experiment after 24 h (*F*_1,28_ = 128, *P* ≤ 0.05) (Fig. S6a), on a per cell basis, intracellular DMSP was not significantly different from −N (*F*_1,28_ = 0.9, *P* = 0.3) (Fig. 5a). The significant diel variability in *E. huxleyi* intracellular DMSP was correlated with the observed changes in cellular Chl*a* (*R* ^2^ = 0.4, *P* ≤ 0.05) (Figs 3c, 5a), suggesting that DMSP was produced at a similar rate as Chl*a* in response to the NO_3_^−^ add‐back. The statistically similar intracellular DMSP at the beginning and end of the experiment was consistent with the constant DMSP concentrations observed in the steady‐state NO_3_^−^ limitation experiment (Fig. S3c). The lack of intracellular DMSP response in *E. huxleyi* to the alleviation of NO_3_^−^ limitation indicated that DMSP was maintained constitutively, independent of nutrient status.

By contrast, after 24 h, +N *T. oceanica* DMSPt was significantly lower than −N DMSPt (*F*_1,28_ = 4, *P* ≤ 0.05) (Fig. S6b) and +N intracellular DMSP rapidly decreased two‐fold (*F*_1,28_ = 22, *P* ≤ 0.05) (Fig. 5b). Specifically, the rapid increase in *T. oceanica* biomass and Chl*a* concentrations in response to the NO_3_^−^ add‐back (Fig. 3d,e) was matched by a similar, rapid decrease in intracellular DMSP (*R* ^2^ = 0.5, *P* ≤ 0.05) (Fig. 5b). We attributed this decrease in *T. oceanica* intracellular DMSP to downregulation of DMSP production to a minimal maintenance rate after alleviation of NO_3_^−^ limitation and subsequent dilution due to cell division (Fig. S7). The +N intracellular DMSP concentration after 24 h was comparable with the observed intracellular DMSP concentrations under steady‐state replete conditions (Table 2). The plasticity of DMSP production by *T. oceanica* in the nonsteady‐state suggested that DMSP was actively regulated in response to the NO_3_^−^ add‐back.

## Discussion

Changes in intracellular DMSP were quantified for a HiDP, *E. huxleyi*, and a LoDP, *T. oceanica*, in a series of monoculture experiments in order to disentangle the different physiological mechanisms of DMSP production in HiDPs and LoDPs. Specifically, to target the two hypothesised types of DMSP regulation (constitutive vs stress‐related), DMSP production was contrasted under metabolically balanced growth conditions (temperatures < *T*_opt_) and metabolically imbalanced growth conditions (temperatures > *T*_opt_ and nutrient limitation). We also directly tested the role of DMSP as a compatible solute in both species under different salinity conditions. Simultaneous physiology measurements (µ, *F*_v_/*F*_m_, and cellular Chl*a*) and estimates of DMSP osmotic contributions provided insight into whether cells were producing DMSP constitutively or as a stress response.

**Figure 6:**
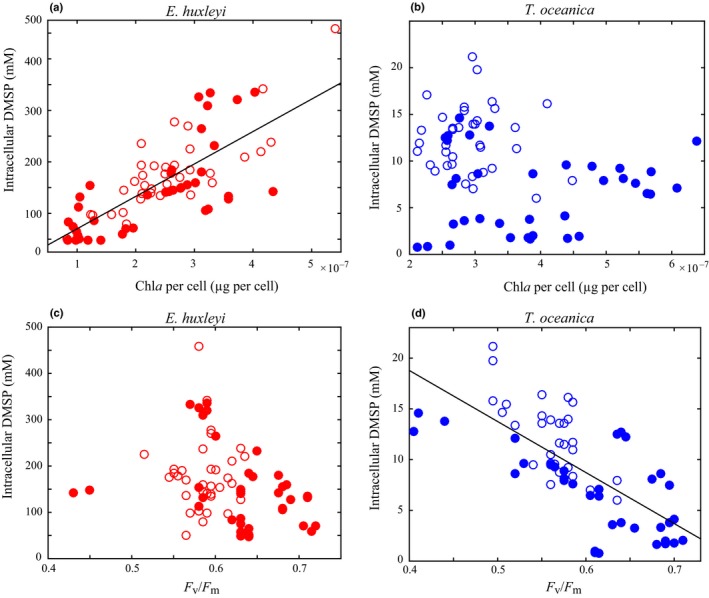
Intracellular dimethylsulfoniopropionate (DMSP) vs Chl*a* per cell and *F*_v_/*F*_m_ for *Emiliania huxleyi* (a, c) and *Thalassiosira oceanica* (b, d) across all steady‐state and nonsteady‐state experiments. Solid circles represent steady‐state experiments. Open circles represent nonsteady‐state experiments. Solid lines are significant linear regressions (*R* ^2^ > 0.6, *P* ≤ 0.05).

Under all conditions, including steady‐state and nonsteady‐state experiments, *E. huxleyi* intracellular DMSP and cellular Chl*a* were significantly positively correlated (Fig. 6a). Despite significant growth limitation, *E. huxleyi* intracellular DMSP did not significantly respond to NO_3_^−^ limitation or temperatures > *T*_opt_, indicating that DMSP production was not altered by these metabolic imbalances (Figs 2a, 5a). Furthermore, *E. huxleyi* intracellular DMSP was not related to *F*_v_/*F*_m_ in any conditions tested (Fig. 6c). Salinity shifts did induce changes in *E. huxleyi* intracellular DMSP (Fig. 2a), but these changes were predicted to be primarily accounted for by shifts in DMSP production to maintain internal osmolarity (Fig. 2c). Low temperature growth (< *T*_opt_) resulted in the only significant change in *E. huxleyi* intracellular DMSP that was independent of salinity changes (Fig. 2a). While this occurrence is the first time that the DMSP response to all of these stressors has been quantified in a single experiment, the general patterns of changes in intracellular DMSP observed here are consistent with previous studies of *E. huxleyi* (Turner *et al.*, 1988; Keller & Korjeff‐Bellows, 1996; Keller *et al.*, 1999a,b; Sunda *et al.*, 2002, 2007; van Rijssel & Gieskes, 2002). It is also important to note the large diurnal variability in cellular Chl*a* and intracellular DMSP (two‐fold) in *E. huxleyi* (Figs 3c, 5a) highlights the significant impact of both sampling time and normalisation factor on resulting measurements of DMSP production in *E. huxleyi*.

The maintenance of high cellular DMSP concentrations by *E. huxleyi*, independent of two contrasting metabolically imbalanced growth conditions (NO_3_^−^ limitation and > *T*_opt_), and the scaling of intracellular DMSP in response to changes in media osmolarity, suggests that DMSP is likely to be a compatible solute in HiDPs. One of the original hypotheses for the DMSP mechanism was that DMSP replaces nitrogen‐containing osmolytes under NO_3_^−^ limitation (Andreae, 1986). Although *E. huxleyi* is known to produce several N‐containing osmolytes (Gebser & Pohnert, 2013; Wordenweber *et al.*, 2018), we found no evidence to support this hypothesis. Intracellular DMSP concentrations in *E. huxleyi* did not increase under NO_3_^−^ limitation, in fact concentrations decreased slightly (Fig. S3c). Intracellular DMSP in *E. huxleyi* did significantly respond to temperatures < *T*_opt_ (Fig. 2a). It has been hypothesised that DMSP may stabilise enzymes and proteins at low temperatures (Nishiguchi & Somero, 1992; Karsten *et al.*, 1996). However, this previous work was conducted at much lower temperatures (6°C) and therefore may not be applicable here. Further work with this HiDP is necessary to determine whether the significant DMSP response under temperatures < *T*_opt_ reflected a shift in compatible solute preference or a secondary role for DMSP.

Maintaining cellular DMSP concentrations in the 100s of mM is believed to require a significant proportion of cellular energy, with a particular demand on methionine synthesis (Stefels, 2000). Given the potential versatility of DMSP as a compatible solute, free radical scavenger, and overflow mechanism, this significant energy demand for synthesis may be justified if HiDPs utilise the multifunctionality of DMSP. Additionally, many HiDPs, including *E. huxleyi,* use a DMSP lyase enzyme to cleave DMSP and produce the membrane permeable gas DMS (Alcolombri *et al.*, 2015). While intracellular DMSP concentrations did not vary significantly across a wide range of environmental conditions, it is still possible that DMSP cycling within the cell changed. Specifically, increased DMSP production would not have been detected if it was accompanied by a corresponding increase in DMSP consumption through DMSP cleavage to DMS or DMSP reaction with ROS. While internal cycling of DMSP is plausible, it is either fairly small in magnitude or very tightly regulated to match production as *E. huxleyi* intracellular DMSP concentrations did not respond to the metabolic imbalances tested here (Fig. 2a). Furthermore, the significant correlation of cellular Chl*a* and DMSP in *E. huxleyi* suggested a coupling of these cellular processes (Fig. 6a). Altogether, these experiments suggest that DMSP is regulated as a constitutive metabolite in this HiDP, likely an essential compatible solute, but also has the potential to serve other physiological roles simultaneously.

Significant changes in *T. oceanica* intracellular DMSP under both balanced and imbalanced growth conditions suggest that *T. oceanica* actively regulated DMSP production in response to stressed growth. In all steady‐state conditions, intracellular DMSP concentrations were significantly correlated with µ (Fig. 2b), and DMSP production was finely tuned to the alleviation of limited growth (Fig. 5b). This finding is consistent with all previous observations of nutrient‐limited LoDP monocultures (*n* = 11), which on average upregulated intracellular DMSP 16‐fold (McParland & Levine, 2019). Intracellular DMSP concentrations were still quantifiable under nonstressed (replete) growth, suggesting that DMSP production is part of the basal metabolism of *T. oceanica*, similar to *E. huxleyi* but at much lower concentrations (Table 2). Unlike *E. huxleyi*, for which DMSP was predicted to contribute up to 100% of total organic osmolarity, intracellular DMSP was predicted to contribute a maximum *c.* 2% of total organic osmolarity in *T. oceanica* (Fig. 2c,d). This small contribution suggests that the regulation of intracellular DMSP by *T. oceanica* is not driven by a compatible solute role. It is possible that DMSP could contribute significantly to cellular osmolarity if maintained within vacuoles or organelles (Lyon *et al.*, 2016), although significant concentrations of DMSP appear to be stored in the cytoplasm (Raina *et al.*, 2017). The plasticity of DMSP production and consistent responses across multiple metabolic conditions suggests that elevated intracellular DMSP concentrations are essential for stressed growth in *T. oceanica*.

A strong negative correlation between intracellular DMSP and *F*_v_/*F*_m_ was observed across all experiments for *T. oceanica* (Fig. 6d), while no relationship was observed for *E. huxleyi* (Fig. 6c). Previous studies have used changes in *F*_v_/*F*_m_ to draw conclusions about the relationship between DMSP production and ROS damage as *F*_v_/*F*_m_ is typically considered an oxidative stress marker (Bucciarelli *et al.*, 2003; Harada *et al.*, 2009; Archer *et al.*, 2010; Darroch *et al.*, 2015). However, *F*_v_/*F*_m_ can also be impacted by the number and configuration of PSII reaction centres. Changes in cellular Chl*a* concentrations and photosystem proteins in response to nutrient status, but independent of oxidative stress, will result in a re‐organisation of PSII, and therefore a change in *F*_v_/*F*_m_ (Butler, 1978; Hailemichael *et al.*, 2016). The small, but significant, changes in *F*_v_/*F*_m_ in our experiments (Δ *F*_v_/*F*_m_ = *c.* 0.05) (Figs 4b, S2) were most likely to be a signature of PSII re‐organisation, not of ROS damage, with the exception of the significant *F*_v_/*F*_m_ decrease in *T. oceanica* under hypersaline conditions (Δ*F*_v_/*F*_m_ = 0.2) which may reflect oxidative stress (Jahnke & White, 2003; Bussard *et al.*, 2017). Therefore, we conclude that the strong correlation between intracellular DMSP and *F*_v_/*F*_m_ in *T. oceanica* is not due to ROS damage, but rather an indirect co‐occurring response to the environmental change. Thus, our results suggest that DMSP in *T. oceanica* is not regulated as an antioxidant, as there was no evidence for ROS damage in temperature and NO_3_^−^ limited growth despite significantly upregulated intracellular DMSP concentrations.

Finally, DMSP synthesis has been proposed to serve as an overflow mechanism to dissipate excess energy during metabolically imbalanced growth (Stefels, 2000). *T. oceanica* intracellular DMSP was significantly upregulated under imbalanced metabolic conditions, but also at low temperatures, when growth was limited but metabolic balance was expected to be maintained (Barton *et al.*, 2018). Therefore, our results suggested that DMSP in *T. oceanica* is not regulated as an overflow mechanism. Of the currently proposed DMSP mechanisms, our findings of DMSP upregulation across different metabolic conditions of stressed growth are not consistent with the osmolyte, antioxidant or overflow mechanisms. Of the currently proposed hypotheses, this leaves the mechanism of a signalling molecule for DMSP production in *T. oceanica* (Seymour *et al.*, 2010; Johnson *et al.*, 2016). Photo‐heterotroph interactions are mediated by infochemicals and are critical for diatoms adapting to different environmental stressors (Amin *et al.*, 2012, 2015; Arandia‐Gorostidi *et al.*, 2017; Durham *et al.*, 2017). Additionally, it has been shown that when concentrated in the phycosphere, very little DMSP is needed to induce chemotaxis by heterotrophic bacteria (Seymour *et al.*, 2010). If DMSP has evolved to serve as a signalling molecule in LoDPs during stressed growth, intracellular DMSP production would be expected to increase across all metabolic conditions of stressed growth, as observed here. This work provides a testable hypothesis for future biochemical work to identify the LoDP mechanism.

To provide a unifying framework for which to interpret 30 yr of conflicting experiments on the cellular function of DMSP, we characterised changes in intracellular DMSP concentrations under metabolically balanced and imbalanced growth and under steady‐state and nonsteady‐state conditions in parallel experiments for a HiDP and LoDP. We found a consistent response of intracellular DMSP to a wide range of environmental stressors for the LoDP (*T. oceanica*) and a different, but also consistent, response for the HiDP (*E. huxleyi*). This work suggests that DMSP is regulated by very different environmental drivers and serves a fundamentally different physiological role for HiDPs and LoDPs. Specifically, our findings suggest that the primary role of DMSP in HiDPs is an essential compatible solute produced constitutively during cell growth. In LoDPs, DMSP is clearly regulated as a stress response and may serve as a signalling molecule.

The significantly different responses of *T. oceanica* and *E. huxleyi* across the conditions tested in this study support the differential regulation of HiDPs and LoDPs proposed by McParland & Levine (2019). While we only used two model species of DMSP producers, the consistent trends observed previously for a wide diversity of HiDP and LoDP strains (McParland & Levine, 2019) provide confidence that the differential DMSP production observed here is representative of other HiDPs and LoDPs. Additionally, the contrasting strategies of DMSP regulation in HiDPs and LoDPs mirror the recent discovery of two DMSP synthesis genes (DSYB and TpMT) that share little homology (Curson *et al.*, 2018; Kageyama *et al.*, 2018). The two genes appear to be differentially present based on HiDP and LoDP taxonomy, suggesting that the HiDP and LoDP phenotypes may have evolved separately (McParland, 2019). More advanced biochemistry methods are needed to investigate this potential evolutionary history and the two cellular mechanisms of DMSP. The first direct monoculture comparisons of HiDPs and LoDPs across multiple metabolic conditions presented here have laid the foundation for future omics‐based approaches to further define the different cellular mechanisms of DMSP in HiDPs and LoDPs.

Previous work has tried to understand variations in *in situ* DMSP production by assuming that DMSP serves a similar physiological function in all DMSP producers. The paradigm of a universal mechanism for DMSP should be reconsidered in the context of this study. Separating DMSP into two different ecological cycles with different underlying genes, regulation and environmental drivers significantly shifts our understanding of *in situ* DMSP cycling. The constitutive regulation of high intracellular DMSP concentrations by HiDPs explains why these producers always dominate *in situ* DMSP production, even in the most nutrient‐limited regions of the ocean where HiDPs are a subdominant community (McParland & Levine, 2019). HiDP dominance of *in situ* DMSP production suggests that HiDPs contribute most significantly to atmospheric release of DMS and climate control at a global scale. By contrast, LoDPs must use limited *in situ* resources to maintain the finely tuned regulation of DMSP production that appears to be essential for stressed growth. If DMSP serves as a signalling molecule or as another related mechanism in LoDPs, then the low concentration of *in situ* DMSP produced by LoDPs is likely to be most important for microbial interactions on the microscale. Future work should consider the importance of differential regulation across DMSP producer taxonomy presented here when quantifying the impact of DMSP on the marine microbial ecosystem, carbon cycling and climate.

## Author contributions

ELM and NML designed the research, analysed results and wrote the manuscript. ELM, AW, KA and MH performed the research. All authors contributed to the writing of the manuscript.

## Supporting information

Please note: Wiley Blackwell are not responsible for the content or functionality of any Supporting Information supplied by the authors. Any queries (other than missing material) should be directed to the *New Phytologist* Central Office.

**Fig. S1** Thermal response curve of *E. huxleyi* and *T. oceanica*.
**Fig. S2** *F*_v_/*F*_m_ measured in *E. huxleyi* and *T. oceanica* steady‐state temperature stress, NO_3_^−^ limitation and salinity stress experiments.
**Fig. S3** Intracellular DMSP measured in *E. huxleyi* and *T. oceanica* steady‐state temperature stress, NO_3_^−^ limitation and salinity stress experiments.
**Fig. S4** *F*_v_/*F*_m_ measured in *T. oceanica* grown at a range of steady‐state NO_3_^−^ concentrations.
**Fig. S5** *F*_v_/*F*_m_ diel cycle for *E. huxleyi* and *T. oceanica* in steady‐state and nonsteady‐state NO_3_^−^ limitation.
**Fig. S6** DMSPt measured in nonsteady‐state *E. huxleyi* and *T. oceanica* after NO_3_^−^ add‐back.
**Fig. S7** Dilution of intracellular DMSP in *T. oceanica* after NO_3_^−^ add‐back due to cell division.
**Notes S1** Description of the midday *F*_v_/*F*_m_ diel feature in *E* *.* *huxleyi* and *T* *.* *oceanica*.
**Table S1** Numbers of cellular biovolumes measurements made for *E. huxleyi* and *T. oceanica* for all experiments.
**Table S2** Assumptions and parameters used to predict the percent contribution of intracellular DMSP to total organic osmolarity.
