# Supplementary material for: Evidence for contrasting roles of dimethylsulfoniopropionate production in Emiliania huxleyi and Thalassiosira oceanica
Source: New Phytol. 2020 Jan 27;226(2):396–409. doi: 10.1111/nph.16374 (PMC7154784; doi:10.1111/nph.16374)
Supplement: Supplementary file 1 — Fig. S1 Thermal response curve of E. huxleyi and T. oceanica. Fig. S2 F v/F m measured in E. huxleyi and T. oceanica steady‐state temperature stress, NO3 − limitation and salinity stress experiments. Fig. S3 Intracellular DMSP measured in E. huxleyi and T. oceanica steady‐state temperature stress, NO3 − limitation and salinity stress experiments. Fig. S4 F v/F m measured in T. oceanica grown at a range of steady‐state NO3 − concentrations. Fig. S5 F v/F m diel cycle for E. huxleyi and T. oceanica in steady‐state and nonsteady‐state NO3 − limitation. Fig. S6 DMSPt measured in nonsteady‐state E. huxleyi and T. oceanica after NO3 − add‐back. Fig. S7 Dilution of intracellular DMSP in T. oceanica after NO3 − add‐back due to cell division. Notes S1 Description of the midday F v/F m diel feature in E . huxleyi and T . oceanica. Table S1 Numbers of cellular biovolumes measurements made for E. huxleyi and T. oceanica for all experiments. Table S2 Assumptions and parameters used to predict the percent contribution of intracellular DMSP to total organic osmolarity. [file NPH-226-396-s001.pdf]

Supporting Information

Article title: Evidence for contrasting roles of dimethylsulfoniopropionate (DMSP) production in *Emiliania huxleyi* and *Thalassiosira oceanica*

Authors: Erin L. McParland, Anna Wright, Kristin Art, Meagan He and Naomi M. Levine

Article acceptance date: 27 November 2019

The following Supporting Information is available for this article:

Notes **S1**: Description of the mid-day F_v/F_m diel feature in *E. huxleyi* and *T. oceanica*.

Table **S1**: Number of cellular biovolumes measurements made for *E. huxleyi* and *T. oceanica* for all experiments.

Table **S2**: Assumptions and parameters used to predict the percent contribution of intracellular DMSP to total organic osmolarity.

Fig. **S1**: Thermal response curve of *E. huxleyi* and *T. oceanica*.

Fig. **S2**: F_v/F_m measured in *E. huxleyi* and *T. oceanica* steady-state temperature stress, NO_3^- limitation and salinity stress experiments.

Fig. **S3**: Intracellular DMSP measured in *E. huxleyi* and *T. oceanica* steady-state temperature stress, NO_3^- limitation and salinity stress experiments.

Fig. **S4**: F_v/F_m measured in *T. oceanica* grown at a range of steady-state NO_3^- concentrations.

Fig. **S5**: F_v/F_m diel cycle for *E. huxleyi* and *T. oceanica* in steady- and non-steady-state NO_3^- limitation.

Fig. **S6**: DMSPt measured in non-steady-state *E. huxleyi* and *T. oceanica* after NO_3^- add-back.

Fig. **S7**: Dilution of intracellular DMSP in *T. oceanica* after NO_3^- add-back due to cell division.

Supporting Information Notes

Notes **S1**: During the non-steady-state NO_3^- add-back experiment, +N *E. huxleyi* and +N and -N *T. oceanica* exhibited a mid-day F_v/F_m minimum that was significantly lower than F_v/F_m at the beginning of the experiment ($p \leq 0.05$) (Main text, Fig. **3**). We tested the reproducibility of this feature by repeating the F_v/F_m measurements in non-steady-state and also steady-state NO_3^- replete and NO_3^- limited conditions. The F_v/F_m diel feature was reproducible in non-steady-state for both *E. huxleyi* and *T. oceanica* (Fig. **S5**). However, the mid-day minimum was absent in *E. huxleyi* under all steady-state NO_3^- conditions (Fig. **S5a**), but still present in *T. oceanica* under all steady-state NO_3^- conditions (Fig. **S5b**). Therefore, the mid-day minimum in F_v/F_m may reflect a response to the non-steady-state NO_3^- add-back only in *E. huxleyi.* In contrast, the mid-day minimum in *T. oceanica* F_v/F_m is likely an inherent component of the species' PSII reaction centers, independent of nutrient status and similar to that observed previously in natural communities of diatoms and picoplankton (Villareal 2004; Mackey et al. 2008).

Supporting Information Tables

Table **S1**: The number of measurements of cell diameter and height made to calculate cellular biovolumes of *T. oceanica* and *E. huxleyi*. Cellular biovolumes did not significantly change across treatments in any of the experiments ($p>0.1$) and therefore the mean cellular biovolume of each species was used to calculate intracellular DMSP for all experimental treatments (experiments 1-4). Error bars represent ± SD.

		E. huxleyi			*T. oceanica*		
		No. counts	Biovolume (μm^3)	±	No. counts	Biovolume (μm^3)	±
Steady-state							
N rep		69	54	15	36	119	36
N lim		71	53	10	50	136	9
14˚C		43	58	18	33	128	36
16˚C		58	63	15	55	113	35
20˚C		57	69	12	33	140	36
23˚C		82	63	19	51	106	22
26˚C		20	63	19	18	120	20
28˚C		21	63	19	19	120	20
Non-steady-state	Timepoint (hr)						
-N	0	21	60	14	32	104	17
-N	1	73	54	10	131	150	30
-N	3	159	54	10	172	132	33
-N	6	149	41	9	180	116	30
-N	9	107	52	10	114	140	33
-N	12	100	41	9	178	125	30
+N	24	120	52	10	98	140	35
+N	1	78	55	13	110	127	22
+N	3	87	65	16	239	134	33
+N	6	115	55	13	223	144	36
+N	9	120	65	16	160	156	36
+N	12	76	52	14	150	131	33
+N	24	119	49	13	175	121	29

Table **S2**: The following parameters were used to predict the percent contribution of intracellular DMSP to total organic osmolarity in each steady-state experiment.

	Treatment	Media Osmolarity	Cellular osmolarity	Na+	K+	Cl-	Ionic osmolarity	% inorganic osmolarity	Organic osmolarity needed	% DMSP contribution to org osmolarity
		(osmol · m^{-3})	(osmol · m^{-3})	(mM)	(mM)	(mM)	(osmol · m^{-3})		(osmol · m^{-3})	
E. huxleyi										
Salinity (‰)	45	1266	1520	396	138	625	1159	76%	361	37%
	40	1124	1349	352	122	555	1029	76%	320	25%
	35	988	1185	309	107	488	904	76%	281	20%
	30	844	1013	264	92	417	772	76%	240	21%
	25	702	842	220	76	347	642	76%	200	24%
Temp (˚C)	14	1013	1216	317	110	500	927	76%	289	112%
	16	1013	1216	317	110	500	927	76%	289	106%
	20	1013	1216	317	110	500	927	76%	289	69%
	23	1013	1216	317	110	500	927	76%	289	50%
	26	1013	1216	317	110	500	927	76%	289	23%
	28	1013	1216	317	110	500	927	76%	289	41%
NO_3^- limitation	replete	1013	1216	317	110	500	927	76%	289	57%
	mid-exponential lim	1013	1216	317	110	500	927	76%	289	46%
	late-exponential lim	1013	1216	317	110	500	927	76%	289	50%
T. oceanica										
Salinity (‰)	50	1408	1690	445	6	595	1045	62%	644	2.13%
	40	1124	1349	355	4	475	835	62%	515	0.69%
	35	988	1185	312	4	417	733	62%	452	0.19%
	25	702	842	222	3	297	521	62%	321	0.16%
Temp (˚C)	14	1013	1216	320	4	428	752	62%	464	2.2%
	16	1013	1216	320	4	428	752	62%	464	1.9%
	20	1013	1216	320	4	428	752	62%	464	1.8%
	23	1013	1216	320	4	428	752	62%	464	1.4%
	26	1013	1216	320	4	428	752	62%	464	0.4%
	28	1013	1216	320	4	428	752	62%	464	0.4%
NO_3^- limitation	replete	1013	1216	320	4	428	752	62%	464	0.8%
	mid-exponential lim	1013	1216	320	4	428	752	62%	464	1.7%
	late-exponential lim	1013	1216	320	4	428	752	62%	464	2.7%

Supporting Information Figures

Fig. **S1**: Thermal response curve of growth rate (μ). μ was calculated with cell concentrations and $\mu_{in\text{-}vivo}$ was calculated with in-vivo fluorescence for *E. huxleyi* (a) and *T. oceanica* (b). Error bars represent ± SD.

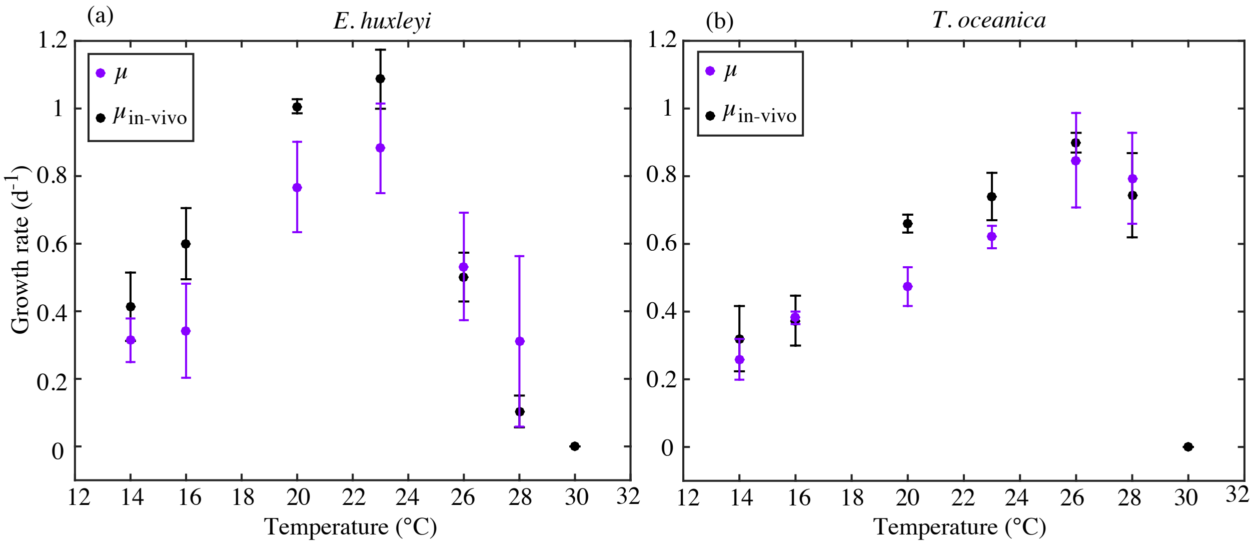

Fig. **S2**: F_v/F_m measured in *E. huxleyi* and *T. oceanica* under steady-state temperature stress (a,b), NO_3^- limitation (c,d) and salinity stress (e,f). Error bars represent ± SD.

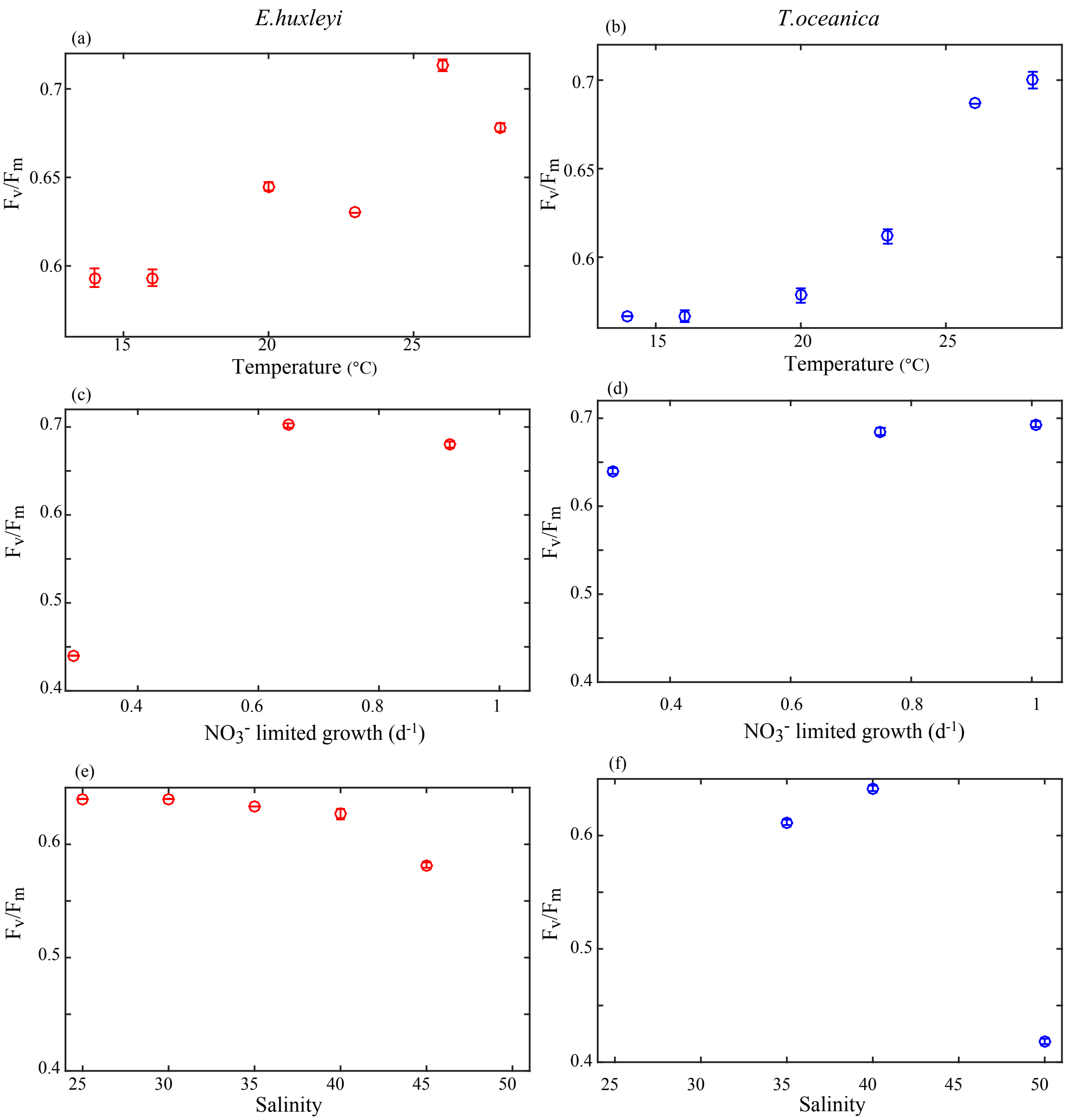

Fig. **S3**: Intracellular DMSP measured in *E. huxleyi* and *T. oceanica* under steady-state temperature stress (a,b), NO_3^- limitation (c,d) and salinity stress (e,f). Error bars represent ± SD.

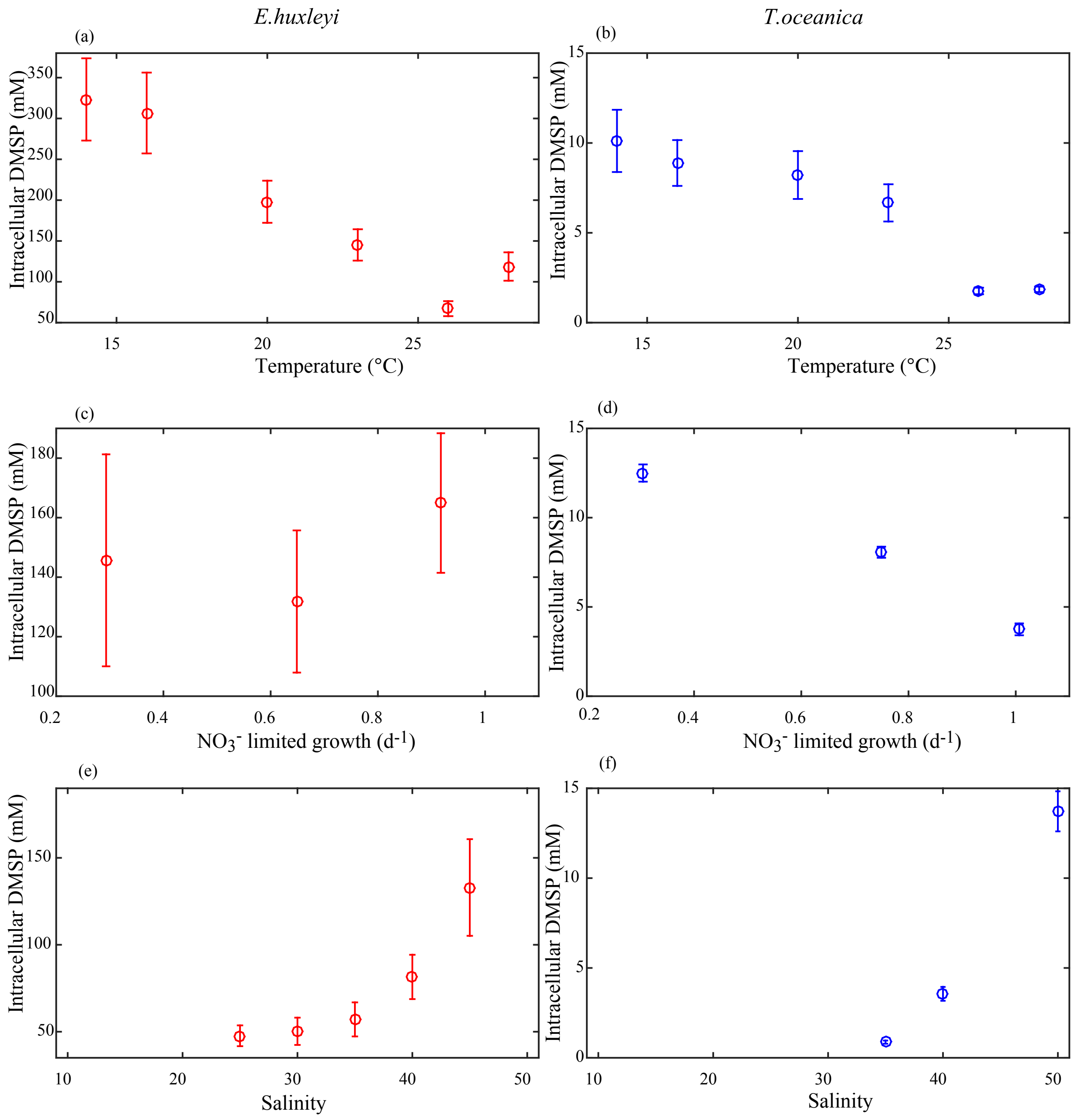

Fig. **S4**: F_v/F_m measured in exponential cultures of *T. oceanica* grown at different steady-state concentrations of NO_3^-. Error bars represent ± SD.

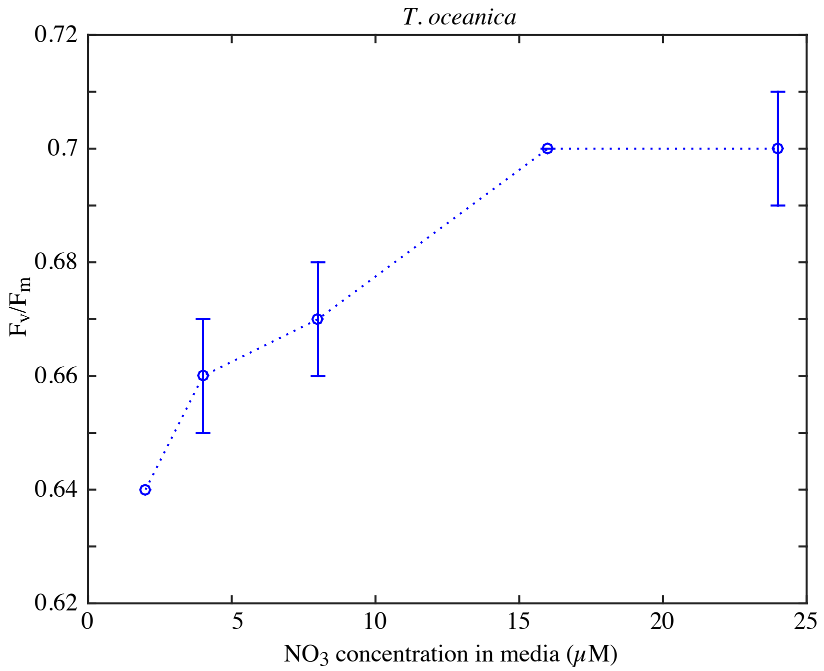

Fig. **S5**: F_v/F_m diel cycle for *E. huxleyi* (a) and *T. oceanica* (b) in steady-state NO_3^- replete (solid black line) and NO_3^- limited (dashed line) conditions and non-steady-state NO_3^- add-back (solid red or blue line). Light cycle (14:10 light:dark) begins at 06:00 h. Experiments began at 07:00 h. Error bars represent ± SD.

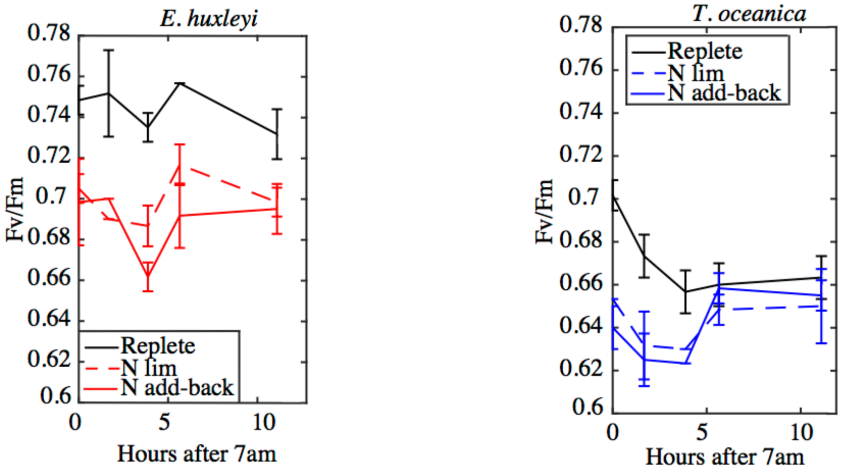

Fig. **S6**: DMSPt after NO_3^- add-back for *E. huxleyi* (a) and *T. oceanica* (b). Solid lines indicate the NO_3^- add-back treatment (+N). The dashed lines represent the control treatment (-N). Grey shading indicates the dark period (14:10 light:dark cycle). Error bars represent ± SD.

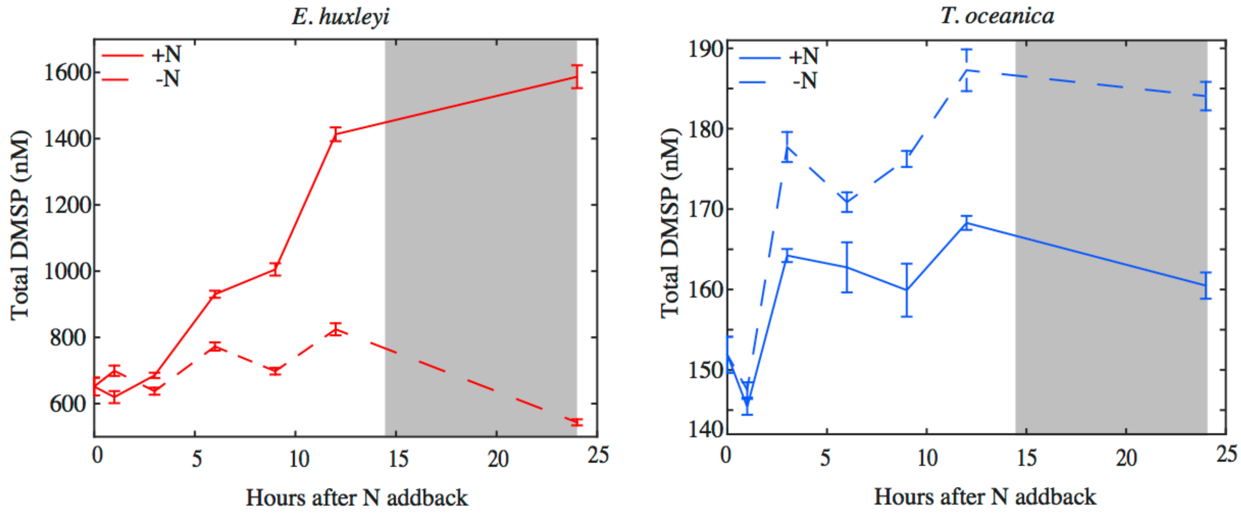

Fig. **S7**: Dilution of intracellular DMSP in *T. oceanica* by cell division. Blue line represents the observed intracellular DMSP (main text, Fig. **5b**). Black line represents intracellular DMSP calculated by holding DMSPt concentration at 0 h constant and dividing by the observed changes in biomass and biovolume. The almost identical results suggest that the rapid decrease after NO_3^- add-back can be primarily attributed to dilution of intracellular DMSP by cell division. Error bars represent ± SD.

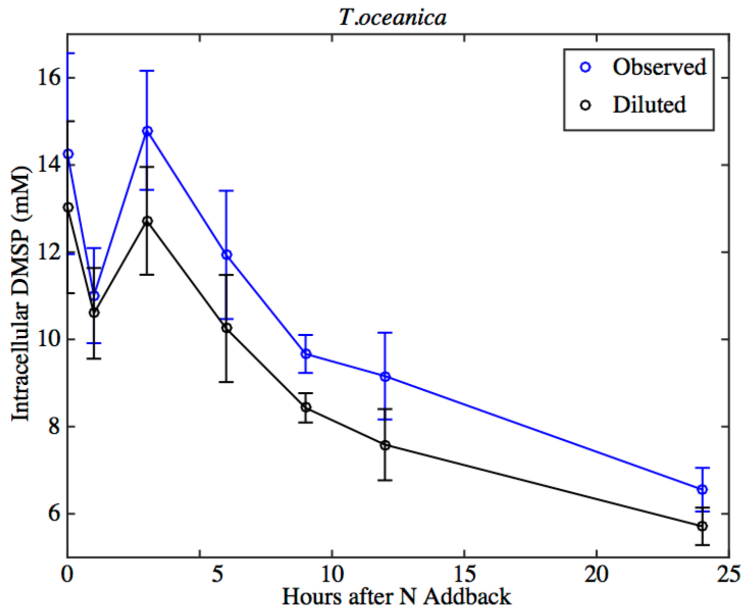

Supplementary References

Mackey, K. R. M., A. Paytan, A. R. Grossman, and S. Bailey. 2008. A photosynthetic strategy for coping in a high-light, low-nutrient environment. Limnol. Oceanogr. **53**: 900–913. doi:10.4319/lo.2008.53.3.0900

Villareal, T. A. 2004. Single-cell pulse amplitude modulation fluorescence measurements of the giant diatom *Ethmodiscus* (Bacillariophyceae). J. Phycol. **40**: 1052–1061. doi:10.1111/j.1529-8817.2004.03208.x
